# High‐Frequency Ultrasound Findings of Nerve Sheath Myxoma in the Instep Skin

**DOI:** 10.1111/srt.70078

**Published:** 2024-10-05

**Authors:** Guiwu Chen, Xiaoling Leng, Haibo Luo, Su Yu, Jiaxin Meng, Xiaomin Liao

**Affiliations:** ^1^ Department of Ultrasound The Tenth Affiliated Hospital of Southern Medical University Dongguan People's Hospital Dongguan China; ^2^ Department of Pathology The Tenth Affiliated Hospital of Southern Medical University Dongguan People's Hospital Dongguan China

Dear Editor,

Nerve sheath myxoma (NSM) is a rare benign peripheral nerve sheath tumor composed of Schwann cells embedded within an abundant myxoid matrix. Typically, NSM presents as a solitary, slow‐growing, asymptomatic mass in the dermis and subcutaneous tissues [1]. Due to their low incidence and lack of symptoms, NSMs are often missed or misdiagnosed. High‐frequency ultrasound facilitates the precise determination of skin tumor depth, revealing intricate ultrasound characteristics and enabling the visualization of blood vessels within, thereby enhancing diagnostic accuracy [2]. Here, we report a case of NSM in the instep skin that was characterized by high‐frequency ultrasound and confirmed by pathological examination.

A 66‐year‐old man presented at our hospital, complaining of discomfort while wearing shoes, and revealed a 40‐year history of a mass in his left foot without any symptoms. Upon physical examination, a protruding mass was observed at the proximal end of the dorsum of the left foot. This mass had a clear boundary, good mobility, no tenderness, and no skin rupture on the surface. Using high‐frequency ultrasound, a solid mass with dimensions of approximately 23 × 11 mm^2^ was discovered within the dermis of the instep skin. This mass exhibited hypoechoic features, defined boundaries, irregular morphology, and internal septae (Figure 1A). Notably, no blood flow signals were detected within the mass (Figure 1B). The patient subsequently underwent surgical resection of the mass, and pathological examination confirmed a diagnosis of NSM. Under hematoxylin and eosin staining, spindle‐shaped cells exhibited nodular proliferation against a backdrop of numerous mucinous substances. The nuclei were small and stained deeply, making nuclear division difficult to observe (Figure 2A). Immunohistochemical staining results were positive for S‐100 and Vim, while CD34, CK, Desmin, SMA, and Ki‐67 were negative (approximately 1%+) (Figure 2B).

**FIGURE 1:**
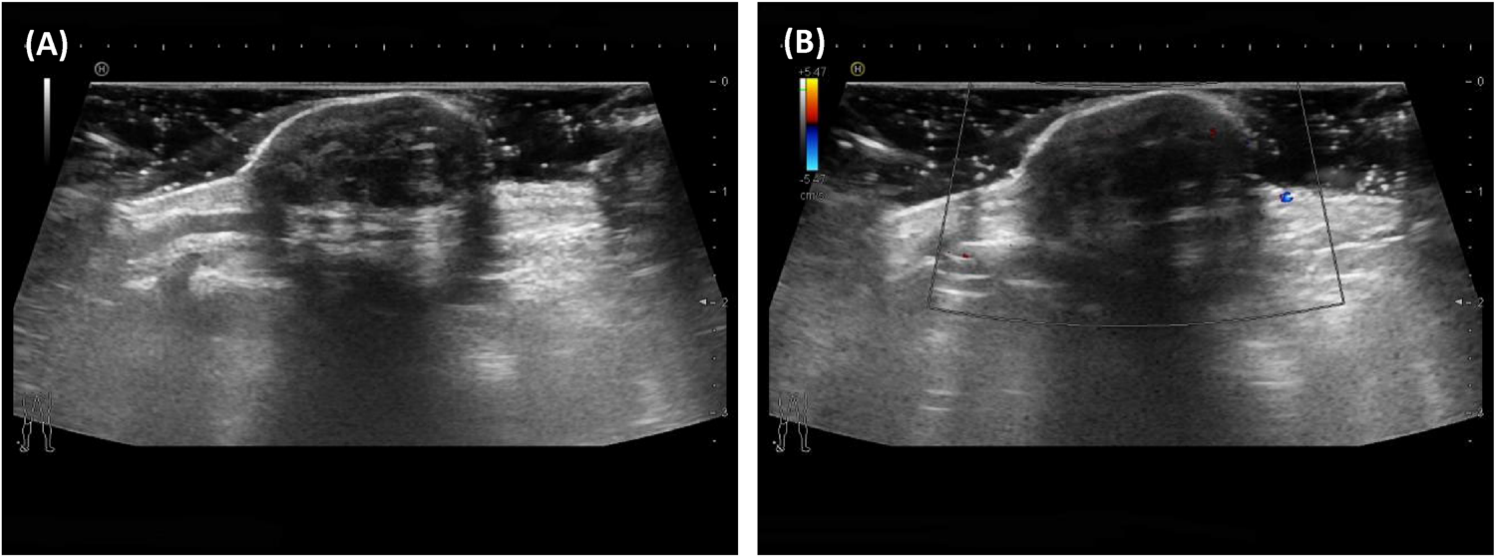
(A) High‐frequency ultrasound show a mass located in the instep skin was hypoechoic, irregular, well‐defined with internal septae. (B) Color Doppler flow imaging showed no blood flow signals within the mass.

**FIGURE 2:**
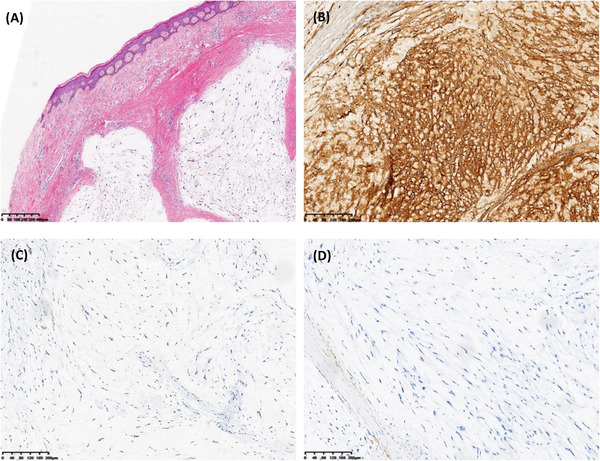
(A) Hematoxylin and Eosin staining showed abundant fluid content of myxoid components and spindled cells separated by fibrous septae. (B), (C), and (D) Immunohistochemical staining showed S‐100 was positive, CK and SMA was negative.

NSM primarily affects young adults between the ages of 30 and 40, regardless of gender. It often originates in the head, neck, spinal canal, and distal extremities. However, due to the lesions' typical locations in the dermis and subcutis, as well as the common practice of physicians to perform surgical excision without imaging, the ultrasound features of NSM have been poorly described in the literature [3]. In our case, high‐frequency ultrasound revealed that the mass was hypoechoic, irregular, and well‐defined with internal septae, while pathological examination confirmed abundant fluid content of myxoid components and spindled cells separated by fibrous septae. Moreover, magnetic resonance imaging is adept at precisely delineating the structure and composition of skin tumors, and when combined with high‐frequency ultrasound, both modalities synergistically enhance the radiological assessment of subcutaneous skin tumors [4]. Currently, the primary treatment for NSM is the complete surgical removal of the mass to prevent recurrence. Neither neoadjuvant nor adjuvant therapy is utilized during treatment.

## Consent

Written informed consent was obtained from the patient to publish this manuscript in accordance with the journal's patient consent policy.

## Conflicts of Interest

The authors declare no conflicts of interest.

## Transparency Statement

We can confirm that this manuscript is an honest, accurate, and transparent account of the case being reported and that no important aspects of the case have been omitted.

## Data Availability

The data used to support the findings of this manuscript are available from the corresponding author upon request.
